# Associations between psychological intervention for anxiety disorders and risk of dementia: a prospective cohort study using national health-care records data in England

**DOI:** 10.1016/S2666-7568(22)00242-2

**Published:** 2023-01

**Authors:** Josh Stott, Rob Saunders, Roopal Desai, Georgia Bell, Caroline Fearn, Joshua E J Buckman, Barbara Brown, Shirley Nurock, Stewart Michael, Paul Ware, Natalie L Marchant, Elisa Aguirre, Miguel Rio, Claudia Cooper, Stephen Pilling, Marcus Richards, Amber John

**Affiliations:** a ADAPT Laboratory, UCL, London, UK; b Centre for Outcomes and Research Effectiveness, Research Department of Clinical, Educational and Health Psychology, UCL, London, UK; c Division of Psychiatry, UCL, London, UK; d Department of Electronic and Electrical Engineering, UCL, London, UK; e MRC Unit for Lifelong Health and Ageing at UCL, UCL, London, UK; f iCope–Camden and Islington Psychological Therapies Services, St Pancras Hospital, London, UK; g Camden & Islington NHS Foundation Trust, St Pancras Hospital, London, UK; h North East London NHS Foundation Trust, London, UK; i Centre for Psychiatry and Mental Health, Queen Mary University of London, London, UK

## Abstract

**Background:**

Meta-analyses support an association between anxiety in older adulthood and dementia. The aim of this study was to use routinely collected health data to test whether treatment of anxiety disorders through psychological intervention is associated with a lower incidence of dementia.

**Methods:**

In this prospective cohort study, data from nationally provided psychological therapy services in England termed Improving Access to Psychological Therapies from 2012 to 2019 were linked to medical records, including dementia diagnoses as defined by the tenth edition of the International Classification of Diseases, up to 8 follow-up years later. Inclusion criteria were as follows: (1) patients who were aged 65 years and older; (2) patients with a probable anxiety disorder; and (3) those with no previous or current diagnosis of dementia. Cox proportional hazards models were constructed to test whether reliable improvement in anxiety following psychological intervention was associated with future dementia incidence. The primary outcome was all-cause dementia and cases were identified using ICD-10 dementia codes from Hospital Episode Statistics, Mental Health Services Dataset, and mortality data. For main analyses, hazards ratios (HRs) are presented.

**Findings:**

Data from 128 077 people aged 65 years and older attending a nationally provided psychological intervention service in England were linked to medical records. 88 019 (69·0%) of 127 064 participants with available gender data were women and 39 585 (31·0%) were men. 111 225 (95·9%) of 115 989 with available ethnicity data were of White ethnicity. The mean age of the sample was 71·55 years (SD 5·69). Fully adjusted models included data from 111 958 people after 16 119 were excluded due to missing data on key variables or covariates. 4510 (4·0%) of 111 958 participants had a dementia diagnosis. The remaining 107 448 (96·0%) were censored either at date of death or when the final follow-up period available for analyses was reached. People who showed reliable improvement in anxiety had lower rates of later dementia diagnosis (3·9%) than those who did not show reliable improvement (5·1%). Reliable improvement in anxiety following psychological intervention was associated with reduced incidence of all-cause dementia (HR 0·83 [95% CI 0·78–0·88]), Alzheimer's disease (HR 0·85 [0·77–0·94]), and vascular dementia (HR 0·80 [0·71–0·90]). Effects did not differ depending on anxiety disorder diagnosis.

**Interpretation:**

Results showed that reliable improvement in anxiety from psychological therapy was associated with reduced incidence of future dementia. There are multiple plausible explanations for this finding and further research is needed to distinguish between these possibilities. Missing data in the sample limit reliability of findings.

**Funding:**

Alzheimer's Society, Medical Research Council, Wellcome Trust, and UCLH National Institute for Health and Care Research Biomedical Research Centre.

## Introduction

Due to the high prevalence and adverse health, economic, and social outcomes of dementia, prevention is a key issue in public health research.^1^ As a result, increasing resources are being invested into investigating potentially modifiable risk factors for dementia. Anxiety disorders, which are common in people older than 65 years,^2^ are one such possible risk factor, and systematic reviews and meta-analyses synthesising data from prospective longitudinal studies have shown that anxiety in midlife (eg, people aged 40–65 years) and older age is associated with increased risk of all-cause dementia,^3^, ^4^ as well as increased risk of developing the two most common forms of dementia: Alzheimer's disease and vascular dementia.^5^ Evidence suggests that the association between common mental health problems and vascular dementia is stronger than the association with other dementia outcomes.^6^

Anxiety consists of various disorders, including generalised anxiety disorder, panic disorder and agoraphobia, social phobia, specific phobias, and health-related or illness anxiety disorder. Obsessive-compulsive disorder (OCD) and post-traumatic stress disorder (PTSD) were also considered to be anxiety disorders until 2013, although they are now separated in the Diagnostic and Statistical Manual of Mental Disorders (version 5). The majority of research investigating anxiety disorders and risk of dementia has focused on generalised anxiety disorder.^3^, ^4^ However, more recent research has also shown that PTSD^7^ and OCD^8^ might also be associated with risk of dementia. Specifically, a recent systematic review and meta-analysis found that PTSD and trauma-related stress were significantly associated with a higher incidence and prevalence of dementia.^9^ Research investigating associations between OCD and dementia is scarce; however, early evidence from a large longitudinal study has shown that people with OCD had a higher incidence of all-cause dementia, Alzheimer's disease, and vascular dementia than did people without OCD.^8^ The underlying mechanisms of the associations between anxiety disorders and dementia are not known, but might be related to biological pathways (eg, alterations in the hypothalamic pituitary adrenal axis), or lifestyle and psychosocial pathways (eg, diet, social support, or exercise). Mechanisms could also differ depending on type of anxiety disorder.

**Research in context**

**Evidence before this study**

Recent systematic reviews and meta-analyses have shown that anxiety is associated with dementia incidence. However, whether effective treatment of anxiety through psychological therapies is associated with a reduction in subsequent dementia incidence remains unknown. We searched PubMed from inception to May 30, 2022, using the following search terms: title/abstract: (anxi*) AND (therap* OR interven*) AND (risk OR odds OR hazard) AND (demen* OR Alzheimer*), with no date or language restrictions. A total of 421 results were generated from the search. After title, abstract, and full-text screening, no relevant articles were identified, highlighting the limited evidence available on this topic.

**Added value of this study**

This study showed that in a sample of 128 077 people who accessed psychological therapies in England from 2012 to 2019, improvement in anxiety symptoms following treatment, and receipt of therapy versus assessment only, were associated with lower incidence of dementia up to 8 years later. The reason for this is unclear. Anxiety might be a risk factor for dementia, which is potentially modifiable through psychological therapies. Alternatively, it is also possible that psychological therapies might be less effective in or less likely to be offered to people with underlying dementia pathology (reverse causality). This study is not able to distinguish between these possibilities.

**Implications of all the available evidence**

These findings are important because older people are currently under-represented in psychological therapies. Older people are five times less likely to access psychological therapies than are young people in the UK, but six times more likely to be prescribed anti-depressant medication. Our findings suggest that improving access to these services for older people should be a key research and policy goal.

The clinically crucial issue of whether anxiety disorders constitute a modifiable risk factor (ie, whether successful intervention for anxiety is associated with reduced risk of dementia) is under-researched. The current study focuses on intervention using psychological therapies. Psychological therapies, including Cognitive Behavioural Therapy, are an effective first-line treatment option for anxiety disorders.^10^ Patients often indicate a preference for psychological interventions for anxiety and other common mental health problems rather than pharmacological approaches,^11^ and psychological interventions can also have value in preventing future relapse of anxiety over a longer period of time.^12^ However, research has not yet addressed whether reductions in symptoms of anxiety during a course of psychological intervention are associated with reduced dementia risk. Whether psychological interventions for specific anxiety disorders are differentially associated with dementia incidence remains unknown.

The primary aim of this study was to test whether effective intervention for anxiety disorders in psychological intervention services offered across England is associated with a reduced risk of future dementia. The secondary aim was to investigate whether these associations differ by type of anxiety disorder or dementia type.

## Methods

### Study design and participants

This prospective cohort study used data from a nationally provided psychological intervention service in England from 2012 to 2019, Improving Access to Psychological Therapies (IAPT). IAPT services offer evidence-based psychological interventions for people in England with common mental health problems, using a stepped care model of delivery.^13^ A standardised minimum dataset is collected in all IAPT services nationally, including information on patient demographic and intervention variables, with intervention variables recorded on a sessional basis.

Data for people seen in IAPT services^14^ were linked to other routinely collected health data in England, including data from the Hospital Episode Statistics (HES) database (admitted patient care, accident and emergency, and outpatient datasets),^15^ HES and Office for National Statistics (ONS) linked mortality data,^16^ and the Mental Health Services Data Set (MHSDS)^17^ using a linkage key provided by National Health Service (NHS) Digital. HES data include information on all inpatient hospital admissions, outpatient appointments, and accident and emergency attendances in England. HES comprise a range of administrative (eg, admission and discharge dates), demographic (eg, age and ethnicity), and clinical (eg, diagnosis and intervention) information about each patient contact with these health services. HES–ONS linked mortality data comprises information about deaths (eg, cause and place of death) of all people ever treated in any England-based hospital (regardless of whether or not they died in hospital). The MHSDS comprises data from specialist mental health services offered in hospitals, outpatient clinics, and in the community.

Participant inclusion criteria at baseline were as follows: (1) patients who were aged 65 years and older; (2) those with a probable anxiety disorder (ie, a score above the clinical threshold for a case outcome measure); and (3) those with no previous or current diagnosis of dementia (identified from HES and MHSDS data) at the time of their treatment in IAPT services. On the basis of national evaluations of IAPT, two or more sessions was considered the minimum requirement for a course of treatment.^14^ Consequently, all patients who received at least two sessions were included in the analytical sample (appendix p 2). For cases in which individuals accessed IAPT services on more than one occasion, the first attendance where a course of treatment was received was used in analyses, to maximise the length of follow-up.

Non-identifiable information was provided by NHS Digital with a legal basis for the anonymisation, and as such this research did not require a research ethics committee review, as per the Governance Arrangements of Research Ethics Committees. This decision was confirmed by the Joint Research Office at UCL and the Health Research Authority in England.

### Procedures

The 7-item Generalised Anxiety Disorder scale (GAD-7) is the anxiety symptom measure routinely used in IAPT.^13^ One of six anxiety disorder specific measures (ADSMs) are recommended for use instead of the GAD-7 for cases in which patients present with specific anxiety disorders (appendix p 5), although there is no ADSM for specific phobias so the GAD-7 is the anxiety-related outcome measure for these and for generalised anxiety disorder. The primary predictor for this study was reliable improvement in anxiety symptoms as measured by the ADSM for the specific anxiety disorder. Reliable improvement refers to a change in symptom scores before and after intervention beyond the threshold for which change might have been likely to occur due to chance alone^18^ (thresholds for each ADSM are presented in the appendix [p 5]). Sensitivity analyses were also run using reliable recovery from anxiety as the primary predictor of incident dementia. Reliable recovery on the appropriate ADSM is defined in this study as both: (1) showing reliable improvement in anxiety scores on the relevant scales, and (2) moving from a score above the clinical threshold for case-level anxiety before intervention to below this threshold at the final intervention appointment (thresholds for caseness are presented in the appendix [p 5]). Additional analyses were also run including anxiety diagnosis type, grouped into eight categories on the basis of the provisional patient diagnosis at referral and informed by analyses of similar data in previous studies.^19^ The categories were as follows: (1) generalised anxiety disorder; (2) OCD; (3) PTSD; (4) phobic anxiety disorders and panic disorders; (5) unspecified anxiety disorders; (6) mixed anxiety and depressive disorders; (7) other (including health-related or illness anxiety, and adjustment disorders); and (8) missing.

Dementia cases were identified using ICD-10 dementia codes from HES, MHSDS, and mortality data, using a case-ascertainment procedure used in previous research.^20^

The primary outcome was all-cause dementia. To minimise effects of undiagnosed dementia during intervention on results, the main analyses excluded any cases of dementia diagnosed within a year of the end of psychological intervention.

Additional prespecified analyses were run using Alzheimer's disease and vascular dementia as the outcome, in line with procedures used in previous research.^20^

### Statistical analysis

Covariates were selected based on variables with known associations with both anxiety disorders and dementia risk. Demographic covariates from the IAPT dataset included age, gender, ethnicity (using codes consistent with UK census), and index of multiple deprivation decile. Clinical covariates were the number of sessions attended in the psychological intervention service, whether the patient reported taking psychotropic medications, whether a patient had a record of cardiovascular disease, presence of comorbid depression, severity of anxiety at the start of intervention, and presence of a long-term health condition. All covariates were taken from the IAPT dataset, except for cardiovascular disease, which was retrieved from HES data.

Missing data were reported for all covariates and the sample with missing data was compared with the sample with complete information on all variables, using χ^2^ tests or *t* tests as appropriate.

Cox proportional hazards models were used to test associations between reliable improvement in anxiety disorders over the course of psychological intervention and incidence of dementia. Participants enter the at-risk period from the last appointment date of psychological intervention. Time-to-event was measured as the years (calculated as days divided by 365) from the end of IAPT intervention course. Participants were censored at the time of death, or when the final follow-up period available for analyses was reached. Missing data were coded as a separate category for categorical covariates with more than 10% missing to maximise sample size in models. The cutoff of 10% was selected because most principled missing data methods suggest that a missing data rate of 10% is inconsequential.^21^ For cases in which missing data was more than 10%, missingness was coded as a separate category so this could be adjusted for in models. This is an approach that has been used frequently in IAPT research.^22^, ^23^, ^24^, ^25^ Three main models were constructed: model 1: unadjusted; model 2: adjusted for demographic covariates; and model 3: adjusted for demographic and clinical covariates. Covariates were based on factors with known associations with psychological therapy outcomes and dementia, which might confound relationships if not included as covariates.

To determine whether models should be stratified by gender, age bands, or ethnicity, models were run using interaction terms for each of these variables. Because no evidence for any such interactions was found, gender, age, and ethnicity were included in models as covariates, rather than as stratifying variables.

To test whether associations between reliable improvement in anxiety and incidence of dementia differed depending on the anxiety disorder type, models were also run including an interaction term between reliable improvement and anxiety disorder type.

To test whether associations between reliable improvement in anxiety symptoms and incidence of dementia differed depending on the type of dementia diagnosis, main models were re-run using Alzheimer's disease and vascular dementia as separate primary outcomes, with no dementia as the reference group. Patients diagnosed with other forms of dementia were excluded from these analyses.

A series of planned sensitivity analyses were also done. First, models were re-run using reliable recovery from anxiety as the main predictor of incident dementia. Models were also re-run using only reliable improvement on the GAD-7 as the primary predictor, and not including other ADSMs, to check whether any bias was introduced by the use of different anxiety measures. To increase confidence that results were not due to reverse causality, models were also re-run excluding cases of dementia diagnosed within 2 years of the end of psychological intervention. Analyses were also re-run excluding patients who reported taking psychotropic medications concurrent with psychological intervention to remove the potential impact of psychotropic medication use.

Sensitivity analyses were also done testing whether completion of a course of treatment (two or more sessions) was associated with lower incident dementia, compared with assessment only (ie, one session only). The control group (people with one session) were selected because they did not complete a course of treatment but did have data available on key covariates (eg, baseline depression and anxiety, and medication status).

Participants who received a subsequent course of treatment were excluded from analyses.

An additional analysis was also run using all-cause mortality as an event and dementia diagnosis as a censored observation. Associations between reliable improvement in anxiety and incident dementia might vary across the country depending on the particular IAPT service delivering the intervention.^26^ To account for this, a mixed effects Weibull survival model was run, including the IAPT service delivering the intervention as a random effect. To account for the non-linear relationship between age and dementia, an additional analysis was done including categorical age bands (65–69, 70–74, 75–79, 80–84, and ≥85 years) as a covariate, instead of as a continuous variable. Finally, an ad-hoc analysis was done using the continuous Patient Health Questionnaire (PHQ)-9 score as a covariate, instead of the binary variable. This was to test whether the main results remained consistent after adjusting for severity of depression (whereby higher PHQ-9 scores represent higher severity). All analyses were done with Stata (version 16).

### Role of the funding source

The funders of the study had no role in study design, data collection, data analysis, data interpretation, or writing of the report, or in the decision to submit the paper for publication.

## Results

IAPT referral data were available from April 1, 2012, to March 31, 2019. The sample comprised a total of 128 077 people aged 65 and older (mean age 71·55 years), excluding people with dementia diagnosed before or up to 1 year after IAPT attendance. The median follow-up available for analyses was 3·12 years (IQR 1·72–4·70). The fully adjusted model included 111 958 people after 16 119 people were excluded due to missing data on key variables and covariates (appendix p 2). More than 10% of data were missing for psychotropic medication use and long-term health conditions. The baseline demographic and clinical characteristics of the sample are outlined in table 1. Characteristics of the analytical sample by reliable improvement status and by dementia status are presented in the appendix (pp 6,8). Specifically, the sample were mainly female (88 019 [69·0%] of 127 604 participants with available gender data) and of White ethnicity (111 225 [95·9%] of 115 989 participants with available ethnicity data). Gender data were self-reported by the patient during IAPT. Approximately 90 782 participants (70·9%) of the 128 077 included in the sample showed reliable improvement in anxiety symptoms, 100 179 (78·3%) of 127 997 had comorbid depression, 52 835 (41·3%) of 128 077 reported a long-term health condition, 62 251 (48·6%) of 128 077 reported taking psychotropic medications, and participants attended 6·19 sessions on average. 71  750 (56·5%) of 127 021 were self-referred to IAPT (either by themselves or by a carer), and 47 672 (37·5%) of 127 021 were referred by a primary health-care provider. Only 7599 (6·0%) of 127 021 were referred into IAPT from other sources. Additionally, 14 140 (11·04%) of the 128 077 people had a linked death record.

**Table 1:** Characteristics of analytical sample of 128 077 participants

		**N (%)**	**Mean (SD)**
**Key variables**			
Anxiety reliable improvement			
	No	37 295 (29·1%)	..
	Yes	90 782 (70·9%)	..
Anxiety reliable recovery			
	No	52 276/127 952 (40·9%)	..
	Yes	75 676/127 952 (59·1%)	..
Dementia diagnosis			
	No	122 650 (95·8%)	..
	Yes	5427 (4·2%)	..
Time to dementia diagnosis, years		..	3·14 (1·48)
**Demographic covariates**			
Gender			
	Male	39 585/127 604 (31·0%)	..
	Female	88 019/127 604 (69·0%)	..
Age		..	71·55 (5·69)
Ethnicity			
	White	111 225/115 989 (95·9%)	..
	Mixed	606/115 989 (0·5%)	..
	Asian	2352/115 989 (2·0%)	..
	Black	1036/115 989 (0·9%)	..
	Chinese	86/115 989 (0·1%)	..
	Other	684/115 989 (0·6%)	..
IMD decile		..	5·99 (2·75)
**Clinical covariates**			
Number of attended contacts		..	6·19 (4·04)
Taking psychotropic medications			
	No	52 114 (40·7%)	..
	Yes	62 251 (48·6%)	..
	Missing	13 712 (10·7%)	..
Cardiovascular disease (at any time)			
	No	75 448/127 981 (59·0%)	..
	Yes	52 533/127 981 (41·0%)	..
Comorbid depression			
	No	27 818/127 997 (21·7%)	..
	Yes	100 179/127 997 (78·3%)	..
Baseline GAD-7 severity		..	13·95 (3·81)
Long-term health condition			
	No	46 398 (36·2%)	..
	Yes	52 835 (41·3%)	..
	Missing	28 844 (22·5%)	..

GAD-7=7-item Generalised Anxiety Disorder scale. IMD=Index of Multiple Deprivation

Of the 128 077 participants, 5427 (4·2%) were diagnosed with dementia at least 1 year after attendance in IAPT. In the 37 295 people who did not show reliable improvement in anxiety, 1918 (5·1%) were diagnosed with dementia in the years following intervention. By contrast, in the 90 782 people who did show reliable improvement in anxiety through intervention, 3509 (3·9%) received a later dementia diagnosis. There were 86 301 people with complete data on all key variables and covariates and 41 776 people who had missing data. There were several key differences between the sample with missing data and the sample with complete information (table 2). People with missing data were significantly more likely to be female, from an ethnic minority background, and from a more deprived area. Additionally, people with missing data were significantly less likely to have an improvement in symptoms of anxiety and more likely to be diagnosed with dementia.

**Table 2:** Missing data analysis

		**Sample with missing data (n=41 776)**	**Sample with complete data (n=86 301)**	**Difference χ^2^ (df), p value; or *t* (df), p value**
**Key variables**				
Anxiety reliable improvement				
	No	13 396 (32·1%)	23 899 (27·7%)	260·89 (1), <0·0001
	Yes	28 380 (67·9%)	62 402 (72·3%)	..
Anxiety reliable recovery				
	No	18 416/41 651 (44·2%)	33 860 (39·2%)	288·36 (1), <0·0001
	Yes	23 235/41 651 (55·8%)	52 441 (60·8%)	..
Dementia diagnosis				
	No	39 480 (94·5%)	83 170 (96·4%)	242·06 (1), <0·0001
	Yes	2296 (5·5%)	3131 (3·6%)	..
**Demographic covariates**				
Gender				
	Male	12 491/41 303 (30·2%)	27 094 (31·4%)	17·34 (1), <0·0001
	Female	28 812/41 303 (69·8%)	59 207 (68·6%)	..
Age		71·54 (5·80)	71·56 (5·63)	−0·62 (128 075), 0·54
Ethnicity				
	White	28 401/29 688 (95·7%)	82 824 (96·0%)	34·20 (5), <0·0001
	Mixed	211/29 688 (0·7%)	395 (0·5%)	..
	Asian	605/29 688 (2·0%)	1747 (2·0%)	..
	Black	247/29 688 (0·8%)	789 (0·9%)	..
	Chinese	23/29 688 (0·1%)	63 (0·1%)	..
	Other	201/29 688 (0·7%)	483 (0·6%)	..
IMD decile		5·82 (2·80)	6·06 (2·73)	−13·87 (123 848), <0·0001
**Clinical covariates**				
Number of attended contacts		6·02 (3·99)	6·27 (4·06)	−10·48 (128 075), <0·0001
Taking psychotropic medications				
	No	12 546/28 064 (44·7%)	39 568 (45·8%)	11·17 (1), 0·0008
	Yes	15 518/28 064 (55·3%)	46 733 (54·2%)	..
Cardiovascular disease (at any time)				
	No	23 946/41 680 (57·5%)	51 502 (59·7%)	57·51 (1), <0·0001
	Yes	17 734/41 680 (42·5%)	34 799 (40·3%)	..
Comorbid depression				
	No	9252/41 696 (22·2%)	18 566 (21·5%)	7·56 (1), 0·0060
	Yes	32 444/41 696 (77·8%)	67 735 (78·5%)	..
Baseline GAD-7 severity		13·89 (3·81)	13·98 (3·80)	−3·88 (128 072), 0·0001
Long-term health condition				
	No	6530/12 932 (50·5%)	39 868 (46·2%)	83·47 (1), <0·0001
	Yes	6402/12 932 (49·5%)	46 433 (53·8%)	..

Data are n (%), n/N (%), or mean (SD), unless otherwise specified. *t* tests were used for continuous variables and χ^2^ test was used for binary or categorical variables. IMD=Index of Multiple Deprivation. GAD-7=7-item Generalised Anxiety Disorder scale.

In the fully adjusted model (model 3; 111 958), 4510 (4·0%) had a dementia diagnosis. The remaining 107 448 (96·0%) were censored either at date of death or when the final follow-up date available for analyses was reached. Reliable improvement in anxiety symptoms during psychological intervention (compared with no reliable improvement) was associated with reduced dementia diagnosis after full adjustment for demographic and clinical covariates (hazard ratio [HR] 0·83 [95% CI 0·78–0·88]; p<0·0001). Older age, Black ethnicity, higher deprivation, attending fewer sessions in IAPT, taking psychotropic medications, cardiovascular disease, and comorbid depression were associated with increased dementia diagnosis (table 3). The Kaplan-Meier plot is presented in the appendix (p 3). The proportional hazards assumption was checked using Schoenfeld residuals and was not violated (χ^2^=25·63, df=17, p=0·082; table 3). Reliable improvement in anxiety symptoms during psychological intervention (compared with no reliable improvement) was also associated with reduced dementia diagnosis in the unadjusted model (model 1) and model adjusted for demographic covariates only (model 2) (model 1: HR 0·77 [95% CI 0·73–0·82], p<0·0001; model 2: HR 0·79 [95% CI 0·74–0·84], p<0·0001).

**Table 3:** Cox proportional hazards models to test associations between reliable improvement in anxiety following psychological intervention and dementia incidence

		**Model 1 (unadjusted; n=128 077)**		**Model 2 (adjusted for demographic factors; n=112 097)**		**Model 3 (adjusted for demographic and clinical factors; n=111 958)**	
		HR (95% CI)	p value	HR (95% CI)	p value	HR (95% CI)	p value
Reliable improvement in anxiety		0·77 (0·73–0·82)	<0·0001	0·79 (0·74–0·84)	<0·0001	0·83 (0·78–0·88)	<0·0001
Gender		..	..	0·83 (0·78–0·89)	<0·0001	0·92 (0·86–0·98)	0·0070
Age		..	..	1·12 (1·12–1·13)	<0·0001	1·11 (1·10–1·11)	<0·0001
Ethnicity							
	White	..	..	1 (ref)	1 (ref)	1 (ref)	1 (ref)
	Mixed	..	..	0·67 (0·42–1·08)	0·10	0·68 (0·42–1·10)	0·12
	Asian	..	..	0·90 (0·71–1·14)	0·40	0·93 (0·73–1·18)	0·54
	Black	..	..	1·38 (1·07–1·78)	0·014	1·44 (1·11–1·86)	0·0054
	Chinese	..	..	0·56 (0·14–2·26)	0·42	0·64 (0·16–2·56)	0·53
	Other	..	..	0·68 (0·42–1·11)	0·12	0·72 (0·44–1·17)	0·18
Deprivation		..	..	0·98 (0·97–0·99)	<0·0001	0·99 (0·98–0·998)	0·016
Number of attended contacts		..	..	..	..	0·99 (0·98–0·99)	0·0015
Taking psychotropic medications							
	No	..	..	..	..	1 (ref)	1 (ref)
	Yes	..	..	..	..	1·19 (1·12–1·27)	<0·0001
	Missing	..	..	..	..	1·18 (1·07–1·30)	0·0010
Cardiovascular disease							
	No	..	..	..	..	1 (ref)	1 (ref)
	Yes	..	..	..	..	2·00 (1·88–2·14)	<0·0001
Comorbid depression							
	No	..	..	..	..	1 (ref)	1 (ref)
	Yes	..	..	..	..	1·13 (1·05–1·22)	0·0013
Baseline GAD-7 severity		..	..	..	..	0·99 (0·98–0·99)	0·048
Long-term health condition							
	No	..	..	..	..	1 (ref)	..
	Yes	..	..	..	..	0·99 (0·93–1·06)	0·80
	Missing	..	..	..	..	1·02 (0·94–1·10)	0·63

Proportional hazards assumption was not violated (χ^2^=25·63, df=17, p=0·082). HR=hazard ratio. GAD-7=7-item Generalised Anxiety Disorder scale.

Results from the model including an interaction term for anxiety disorder diagnosis type showed that after accounting for diagnosis type there was still a significant association between reliable improvement in anxiety symptoms and dementia incidence (table 4; HR 0·78 [95% CI 0·66–0·93], p=0·0039). There were no significant interactions of any anxiety disorder diagnosis type with dementia incidence (with generalised anxiety disorder as the reference category). Additionally, this model showed that there were no significant interactions between reliable improvement and anxiety disorder (table 4). The proportional hazards assumption was violated for this analysis (χ^2^=52·71, df=31, p=0·0088). This was due to non-proportional hazards of PTSD and gender. As anxiety disorder type is a key variable in this analysis, participants with PTSD were excluded from the analysis. Gender was included as a strata variable to allow a distinct baseline hazard for each stratum. Results from this model remained consistent, showing that there were no significant interactions between reliable improvement and anxiety disorder (table 4).

**Table 4:** Interaction effects between anxiety diagnosis type and reliable improvement following psychological intervention on dementia incidence

		Model 1*		**Model 2**†	
		HR (95% CI)	p value	HR (95% CI)	p value
Reliable improvement in anxiety		0·78 (0·66–0·93)	0·0039	0·78 (0·67–0·93)	0·0040
Anxiety diagnosis type					
	Generalised anxiety	1 (ref)	1 (ref)	1 (ref)	1 (ref)
	OCD	0·60 (0·27–1·36)	0·22	0·60 (0·27–1·35)	0·22
	PTSD	0·61 (0·32–1·14)	0·12	..	..
	Phobic anxiety and panic	0·92 (0·69–1·23)	0·58	0·92 (0·69–1·23)	0·58
	Unspecified anxiety	0·67 (0·25–1·81)	0·43	0·67 (0·25–1·81)	0·43
	Mixed anxiety and depression	1·02 (0·85–1·22)	0·86	1·02 (0·85–1·22)	0·87
	Other	0·95 (0·81–1·13)	0·57	0·95 (0·81–1·13)	0·57
	Missing	0·96 (0·82–1·13)	0·64	0·96 (0·82–1·13)	0·64
Interaction: reliable improvement and anxiety diagnosis type					
	OCD	1·68 (0·64–4·44)	0·29	1·69 (0·64–4·45)	0·29
	PTSD	1·77 (0·85–3·65)	0·13	..	..
	Phobic anxiety and panic	0·96 (0·68–1·36)	0·82	0·96 (0·68–1·36)	0·82
	Unspecified anxiety	1·72 (0·53–5·56)	0·36	1·73 (0·53–5·57)	0·36
	Mixed anxiety and depression	1·09 (0·88–1·36)	0·43	1·09 (0·88–1·36)	0·43
	Other	1·10 (0·90–1·35)	0·33	1·10 (0·90–1·35)	0·33
	Missing	1·00 (0·82–1·22)	0·99	1·00 (0·82–1·21)	0·99
Gender		0·92 (0·86–0·98)	0·0070	..	..
Age		1·11 (1·10–1·11)	<0·0001	1·11 (1·10–1·11)	<0·0001
Ethnicity					
	White	1 (ref)	1 (ref)	1 (ref)	1 (ref)
	Mixed	0·69 (0·43–1·10)	0·12	0·70 (0·43–1·13)	0·14
	Asian	0·93 (0·73–1·17)	0·53	0·91 (0·72–1·56)	0·44
	Black	1·44 (1·11–1·86)	0·0056	1·43 (1·10–1·85)	0·0076
	Chinese	0·64 (0·16–2·55)	0·52	0·64 (0·16–2·58)	0·53
	Other	0·72 (0·44–1·17)	0·18	0·72 (0·44–1·18)	0·19
Deprivation		0·99 (0·98–0·99)	0·013	0·99 (0·98–0·99)	0·017
Number of attended contacts		0·99 (0·98–0·99)	0·0020	0·99 (0·98–0·99)	0·0020
Taking psychotropic medications					
	No	1 (ref)	1 (ref)	1 (ref)	1 (ref)
	Yes	1·19 (1·12–1·27)	<0·0001	1·19 (1·12–1·27)	<0·0001
	Missing	1·18 (1·07–1·31)	·0007	1·18 (1·07–1·30)	0·0010
Cardiovascular disease					
	No	1 (ref)	1 (ref)	1 (ref)	1 (ref)
	Yes	2·00 (1·88–2·14)	<0·0001	2·00 (1·87–2·13)	<0·0001
Comorbid depression					
	No	1 (ref)	1 (ref)	1 (ref)	1 (ref)
	Yes	1·12 (1·03–1·21)	0·0061	1·12 (1·03–1·21)	0·0050
Baseline GAD-7 severity		0·99 (0·98–1·00)	·056	0·99 (0·98–1·00)	0·066
Long-term health condition					
	No	1 (ref)	1 (ref)	1 (ref)	1 (ref)
	Yes	0·99 (0·92–1·06)	0·71	0·99 (0·93–1·06)	0·87
	Missing	1·02 (0·94–1·10)	0·68	1·03 (0·95–1·11)	0·53

HR=hazard ratio. OCD=obsessive compulsive disorder. PTSD=post-traumatic stress disorder. GAD-7=7-item Generalised Anxiety Disorder scale.

* Model 1, fully adjusted model including interaction term between anxiety diagnosis type and reliable improvement: proportional hazards assumption was violated (χ^2^=52·71, df=31, p=0·0088).

† Model 2, as in model 1, excluding PTSD and including gender as a strata variable: proportional hazards model was not violated (χ^2^=39·99, df=28, p=0·066).

Results from Cox proportional hazards models showed that reliable improvement in anxiety symptoms following psychological intervention was associated with reduced incidence of Alzheimer's disease, after excluding all other forms of dementia (HR 0·85 [95% CI 0·77–0·94]; p=0·0009). The results were consistent when using vascular dementia as the primary outcome (HR 0·80 [95% CI 0·71–0·90]; p<0·0003; table 5). The effect sizes produced for Alzheimer's disease and vascular dementia were similar. The Kaplan-Meier plots are presented in the appendix (p 4). The proportional hazards assumption was not violated for either model (Alzheimer's disease: χ^2^=18·74, df=17, p=0·34; vascular dementia: χ^2^=20·76, df=17, p=0·24).

**Table 5:** Cox proportional hazards models to test associations between reliable improvement in anxiety and dementia subtypes

		**Alzheimer's disease (n=109 415)**		**Vascular dementia (n=108 693)**	
		HR (95% CI)	p value	HR (95% CI)	p value
Reliable improvement in anxiety		0·85 (0·77–0·94)	0·0009	0·80 (0·71–0·90)	0·0003
Gender		1·05 (0·95–1·16)	0·30	0·78 (0·69–0·88)	<0·0001
Age		1·11 (1·11–1·12)	<0·0001	1·11 (1·10–1·12)	<0·0001
Ethnicity					
	White	1 (ref)	1 (ref)	1 (ref)	1 (ref)
	Mixed	0·99 (0·55–1·80)	0·98	0·58 (0·22–1·54)	0·27
	Asian	0·94 (0·65–1·35)	0·74	0·90 (0·58–1·41)	0·65
	Black	1·40 (0·94–2·09)	0·096	1·77 (1·14–2·77)	0·012
	Chinese	0·66 (0·09–4·72)	0·68	..	..
	Other	0·61 (0·27–1·36)	0·23	0·63 (0·24–1·69)	0·36
Deprivation		0·99 (0·97–1·01)	0·20	0·97 (0·95–0·99)	0·0012
Number of attended contacts		0·98 (0·97–0·99)	0·0098	0·98 (0·96–0·99)	0·0032
Taking psychotropic medications					
	No	1 (ref)	1 (ref)	1 (ref)	1 (ref)
	Yes	1·12 (1·02–1·23)	0·019	1·31 (1·16–1·48)	<0·0001
	Missing	1·07 (0·92–1·24)	0·38	1·21 (1·00–1·46)	0·049
Cardiovascular disease					
	No	1 (ref)	1 (ref)	1 (ref)	1 (ref)
	Yes	1·47 (1·34–1·62)	<0·0001	4·31 (3·72–4·99)	<0·0001
Comorbid depression					
	No	1 (ref)	1 (ref)	1 (ref)	1 (ref)
	Yes	0·93 (0·83–1·03)	0·17	1·30 (1·12–1·52)	0·0007
Baseline GAD-7 severity		0·99 (0·98–1·01)	0·25	1·00 (0·98–1·01)	0·58
Long-term health condition					
	No	1 (ref)	1 (ref)	1 (ref)	1 (ref)
	Yes	0·87 (0·79–0·97)	0·010	0·98 (0·86–1·11)	0·75
	Missing	1·00 (0·89–1·12)	0·98	0·94 (0·80–1·10)	0·42

Proportional hazards assumption was not violated (Alzheimer's disease: χ^2^=18·74, df=17, p=0·34; vascular dementia: χ^2^=20·76, df =17, p=0·24). HR=hazard ratio. GAD-7=7-item Generalised Anxiety Disorder scale.

Models including reliable recovery from anxiety as the primary predictor of incident dementia were consistent with the main models (HR 0·83 [95% CI 0·78–0·88], p<0·0001; appendix p 10). Models showed that reliable improvement in GAD-7 was also associated with reduced incident dementia (HR 0·85 [95% CI 0·80–0·91], p<0·0001; appendix p 12). The model excluding cases of dementia diagnosed within 2 years of the end of psychological intervention was also consistent with main models, showing significant associations between reliable improvement in anxiety and reduced incidence of dementia (HR 0·89 [95% CI 0·83–0·96], p=0·0027; appendix p 14). Results from the model including only patients who reported not taking psychotropic medications at the time of intervention were consistent with main models, with significant associations between reliable improvement in anxiety and reduced dementia incidence (HR 0·87 [95% CI 0·79–0·97], p=0·012; appendix p 16). The model testing whether completion of a course of treatment (n=70 292) was associated with incident dementia compared with assessment only (n=31 457) showed that completing a course of treatment in IAPT was significantly associated with reduced incidence of dementia (HR 0·79 [95% CI 0·74–0·83], p<0·0001; appendix p 18). The fully adjusted model including all-cause mortality as an event and dementia records as censored observations showed that reliable improvement in anxiety was significantly associated with lower mortality (HR 0·84 [95% CI 0·81–0·88], p<0·0001; appendix p 20). Results also remained consistent after including a random effect for the IAPT service (HR 0·83 [95% CI 0·78–0·89], p<0·0001; appendix p 22). Additionally, the association between reliable improvement and dementia remained consistent after including categorical age bands in the model as a covariate (HR 0·82 [95% CI 0·77–0·87], p<0·0001; appendix p 24). Finally, main results also remained consistent after using the continuous PHQ-9 measure as a covariate to account for severity of symptoms (HR 0·84 [95% CI 0·79–0·89], p<0·0001). There was also a significant association between depression severity at the start of treatment and dementia incidence, although the effect size was small (HR 1·02 [95% CI 1·01–1·02], p<0·0001; appendix p 26).

## Discussion

This study has demonstrated that reliable improvement in anxiety symptoms following psychological intervention is associated with reduced incidence of all-cause dementia, Alzheimer's disease, and vascular dementia (compared with no reliable improvement). There was no evidence of differential effects by anxiety disorder. The findings were consistent when using alternative metrics of intervention outcome (reliable recovery and GAD-7 scale), after accounting for service-level variation, and after excluding cases in which dementia was diagnosed within 2 years of the end of the intervention. Results also showed that completing a course of treatment in IAPT was significantly associated with reduced dementia incidence, compared with assessment only. Sensitivity analyses also showed that reliable improvement in anxiety was significantly associated with reduced mortality, suggesting that results from main analyses are unlikely to be due to the competing risk of mortality.

It is important to note that these results are not causal and there are multiple plausible explanations for this link, which this study cannot distinguish between. Firstly, there might be a direct effect of symptom change in anxiety during psychological interventions on dementia risk reduction. There are multiple plausible mechanisms which could underlie the association between change in anxiety symptoms during psychological interventions and dementia incidence. Specifically, this association could operate through a range of distinct biological (eg, cardiometabolic factors^27^), psychosocial (eg, repetitive negative thinking^28^), and lifestyle (eg, physical activity^29^) pathways. Alternatively, the associations between improvement in anxiety symptoms through psychological therapies and dementia incidence might be related to residual confounding—for example, vascular pathology.^30^ In an attempt to account for confounding, cardiovascular disease and other important demographic and clinical factors were included in analytical models. Observed results might potentially be due to reverse causality. Specifically, as there were no cognitive data available at baseline, underlying dementia neuropathology (before dementia diagnosis) might affect response to psychological interventions. To explore this possibility, models excluded dementia cases diagnosed within 1 (main models) and 2 (supplementary models) years from the end of psychological intervention. However, this does not eliminate the reverse causality as a potential explanation of results, especially given the smaller effect size observed in the 2-year model. Psychological therapies might potentially be less effective in or less likely to be offered to people with underlying dementia pathology.^31^

Mild behavioural impairment refers to the emergence of neuropsychiatric symptoms (including anxiety symptoms) in people older than 50 years, and is considered to be a marker of dementia risk.^32^ Understanding factors that are associated with progression to dementia in people with mild behavioural impairment is an important goal for research. In this study, anxiety disorders could not be differentiated from mild behavioural impairment. Therefore, a further plausible explanation for these findings is that effective intervention to reduce neuropsychiatric symptoms in people with mild behavioural impairment might be associated with reduced risk of progression to dementia in this at-risk population. People with mild behavioural impairment might be less likely to have an improvement in anxiety symptoms during treatment than those without this condition. Future research that can clearly distinguish between anxiety disorders and mild behavioural impairment is needed to better understand these possibilities.

These results are consistent with some previous studies which used similar designs to test whether intervention to improve other dementia risk factors is associated with decreased dementia diagnoses. For example, one study showed that in a population of older people with hearing loss, the risk of dementia diagnosis was significantly lower in those who used hearing aids, compared with those who did not.^33^ Effect sizes reported were similar. The literature on the associations between interventions to improve a range of dementia risk factors and dementia risk is outlined in the *Lancet* Commission on dementia prevention, intervention, and care.^1^, ^34^

Strengths of this study are the large sample of older people accessing psychological therapies included in analyses (n=128 077), and the availability of high-quality data from all psychological intervention services in every health authority area of England.

Despite the large sample size, there is a limitation to what we can firmly conclude from the results because of the issue of reverse causality. Although dementia cases in the 1 and 2 years after therapy were excluded, without a measurement of cognitive function taken prior to intervention, it is not possible to fully account for the influence of cognitive deficits that are short of a dementia diagnosis on intervention outcomes. Additionally, although important demographic and clinical factors were adjusted for in the models, data were not available on other important variables, including education, genetic risk, family history, vascular pathology, and physical activity. Analyses also could not account for interventions offered after completion of psychological therapies. Patients who did not respond to psychological therapies might be more likely to be offered antidepressant medications than patients whose symptoms improved. Additionally, data were not available on the type of psychotropic medication used, meaning more detailed analyses about psychotropic medication type were not possible. A further limitation of this study was the exclusion of participants with incident dementia within 1 year (2 years in sensitivity analysis) of the end of psychological intervention. The use of a 1-year cutoff is ultimately arbitrary, but was selected to minimise issues of reverse causality, while also maximising available sample size.

Although all analyses were pre-planned and hypothesis-led and the sample size was large, multiple statistical tests and models were fitted, which might contribute towards a higher chance of type 1 error. Results should be interpreted in the context of this limitation. Additionally, a further limitation is that although therapies were evidence based and delivered according to standardised protocols developed in randomised controlled trials, the specific therapies offered to individuals and for how long varied across individuals.

Additionally, the study sample comprised only those who sought and received psychological therapy services for anxiety symptoms. Evidence suggests that ethnic minority groups experience inequalities in accessing and receiving treatment in IAPT services.^35^ The majority of the sample from this study were White, with only 4·1% from ethnic minority backgrounds. The Office for National Statistics used IAPT data, 2011 Census records, and the UK Household Longitudinal Survey to estimate rates of access to IAPT among people with a probable common mental health disorder.^36^ They reported that older people, men, people born outside the UK, people for whom English is not a first language, people from ethnic minority backgrounds (particularly those from Asian backgrounds), people with a religion, and disabled people were under-represented in IAPT services. As such, it is likely that the results reported in this study are not representative or generalisable to the wider UK population. Further research is required to understand these associations within under-represented groups.

Furthermore, there were no high quality data on duration of depression or anxiety, or number of episodes experienced. This means that this information could not be considered in the analyses. Additionally, physician interviews for anxiety symptoms are not available in IAPT data, meaning analyses rely on self-report scale scores of anxiety. The conclusions that can be drawn from this study are also limited by missing data. Analyses revealed that the sample with missing data did differ significantly from the sample without missing data on key variables and covariates. As such, the data are either missing at random or missing completely at random. Additionally, although data are available over the course of 8 years, the median follow-up available for analyses was 3·12 years, meaning dementia outcomes over a longer period of time could not be examined. Results should be interpreted in the context of these limitations.

Finally, it should be noted that ADSMs were gradually introduced into IAPT; therefore, in some cases, GAD-7 was used for anxiety disorders other than generalised anxiety.

Psychological therapies are an evidence-based treatment option for older people with anxiety disorders, and are recommended by UK National Institute for Health and Care Excellence guidelines^37^ and WHO.^38^ Research shows that older people are more likely to benefit from intervention and less likely to discontinue once intervention is initiated than are younger people.^22^ Additionally, these results suggest that improving symptoms of anxiety in psychological therapies might be associated with longer-term impacts on dementia incidence. However, older people remain under-represented in psychological intervention services compared with younger people. Older people are five times less likely to access psychological therapies than are young people in the UK, but six times more likely to be prescribed antidepressant medication.^39^ Evidence from this research suggests that improving access to evidence-based psychological therapies for older people with anxiety could contribute to dementia risk reduction.

As a next step, programmes to increase access to psychological interventions in older people could be developed and evaluated to test whether in addition to expected benefits in psychological health, there are also benefits for cognitive function. Future research should focus on testing associations between improvement in anxiety symptoms and dementia incidence within under-represented groups, including people from minority ethnic backgrounds. Future research should also focus on whether psychological therapies for anxiety can slow rate of cognitive decline in people already living with dementia.

Overall, this research shows that improving anxiety symptoms in psychological therapies is associated with a reduced incidence of all-cause dementia, Alzheimer's disease, and vascular dementia in older people, with a follow-up period of up to 8 years. Notably, these results are not causal and multiple potential pathways could underlie this association. Future research should seek to distinguish between these possibilities. Most of the study sample were female and White; however, further research is needed to understand these associations within under-represented groups.

## Data sharing

The data fields used for the study can be accessed via the NHS Digital data dictionary: https://www.datadictionary.nhs.uk/. All data used for this study are available upon successful application to NHS Digital via the Data Access Request Service: https://digital.nhs.uk/services/data-access-request-service-dars.

## Declaration of interests

JS, MiR, AJ, NLM, and GB report grant funding from the Alzheimer's Society and MaR reports grant funding from the UK Medical Research Council. JEJB reports receiving grant funding from the Wellcome Trust, the Royal College of Psychiatrists, and UCL Institute of Mental Health. RS reports receiving funding from NHS England. AJ reports fellowship funding from Alzheimer's Research UK. NLM reports funding from the EU's Horizon 2020 research and innovation programme and the Alzheimer's Society.
