# Supplementary material for: Associations between psychological intervention for anxiety disorders and risk of dementia: a prospective cohort study using national health-care records data in England
Source: Lancet Healthy Longev. 2023 Jan;4(1):e12–22. doi: 10.1016/S2666-7568(22)00242-2 (PMC10570142; doi:10.1016/S2666-7568(22)00242-2)
Supplement: Supplementary appendix [file mmc1.pdf]

THE LANCET
Healthy Longevity

Supplementary appendix

This appendix formed part of the original submission and has been peer reviewed. We post it as supplied by the authors.

Supplement to: Stott J, Saunders R, Desai R, et al. Associations between psychological intervention for anxiety disorders and risk of dementia: a prospective cohort study using national health-care records data in England. *Lancet Healthy Longev* 2022; published online Dec 9. https://doi.org/10.1016/S2213-2600(22)00260-0.

Supplementary Materials

Supplementary Figure 1: Study flow chart.

Supplementary Figure 2: Kaplan Meier plot.

Supplementary Table 1: Thresholds for caseness and reliable change for Anxiety Disorder Specific Measures (ADSMs).

Supplementary Table 2: Characteristics of analytic sample by reliable improvement status.

Supplementary Table 3: Characteristics of analytic sample by dementia status.

Supplementary Table 4: Cox proportional hazards models to test associations between reliable recovery from anxiety following psychological therapy and dementia incidence.

Supplementary Table 5: Cox proportional hazards models to test associations between reliable improvement in GAD-7 following psychological therapy and dementia incidence.

Supplementary Table 6: Cox proportional hazards models to test associations between reliable improvement in anxiety following psychological therapy and dementia incidence, excluding dementia cases diagnosed within 2 years after IAPT.

Supplementary Table 7: Cox proportional hazards models to test associations between reliable improvement in anxiety following psychological therapy and dementia incidence, excluding people taking psychotropic medications.

Supplementary Table 8: Cox proportional hazards models to test associations between completion of a course of treatment in IAPT (2+ sessions) and dementia incidence.

Supplementary Table 9: Cox proportional hazards models including all-cause mortality as an event and dementia diagnosis as censored observations.

Supplementary Table 10: Mixed effects Weibull survival model testing associations between reliable improvement in anxiety and dementia incidence, including IAPT service delivering intervention as a random effect.

Supplementary Table 11: Cox proportional hazards models including categorical age bands.

Supplementary Table 12: Cox proportional hazards models including continuous PHQ-9 score.

Supplementary Figure 1: Study flow chart.

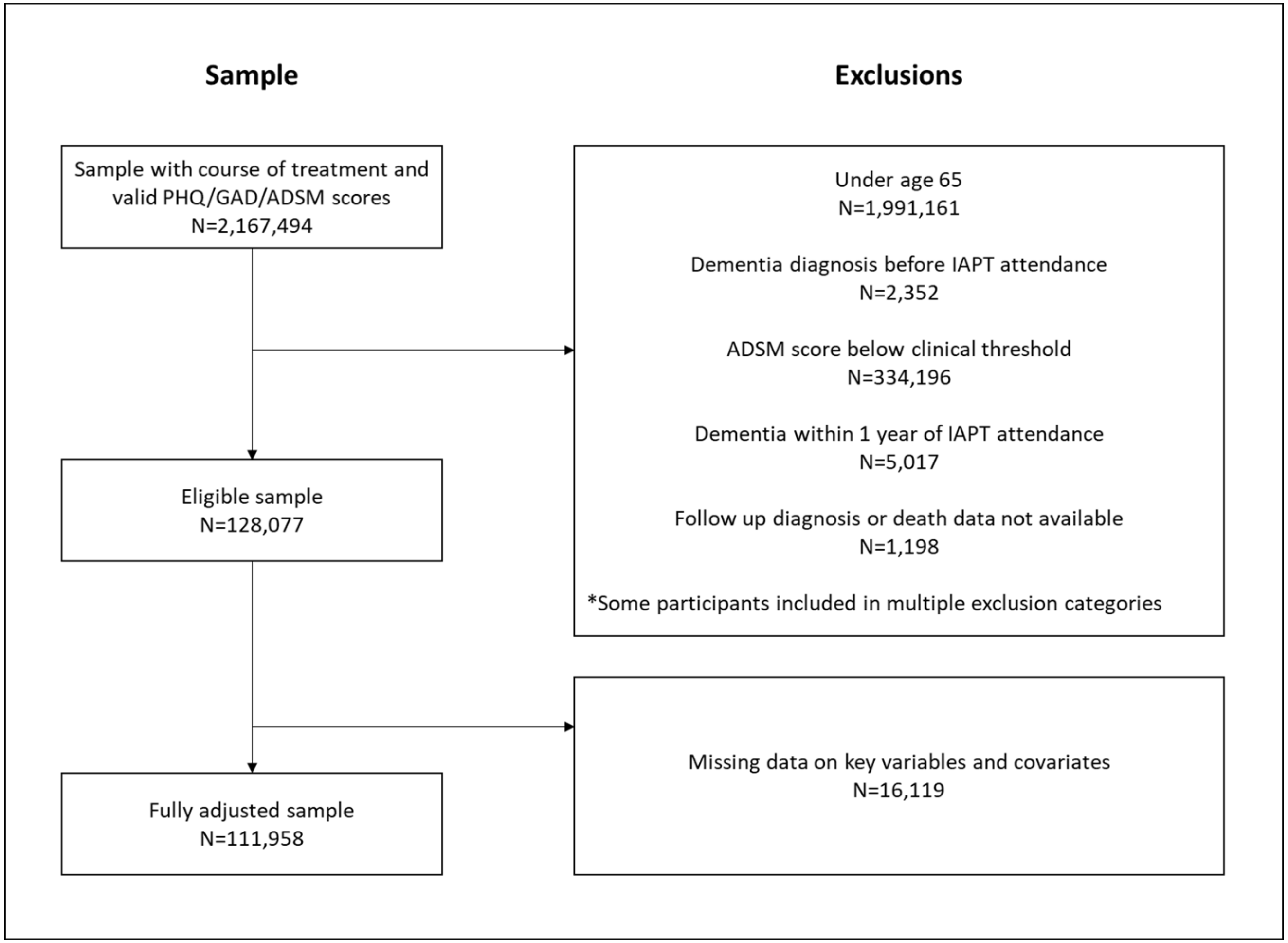

Supplementary Figure 2: Kaplan Meier plot.

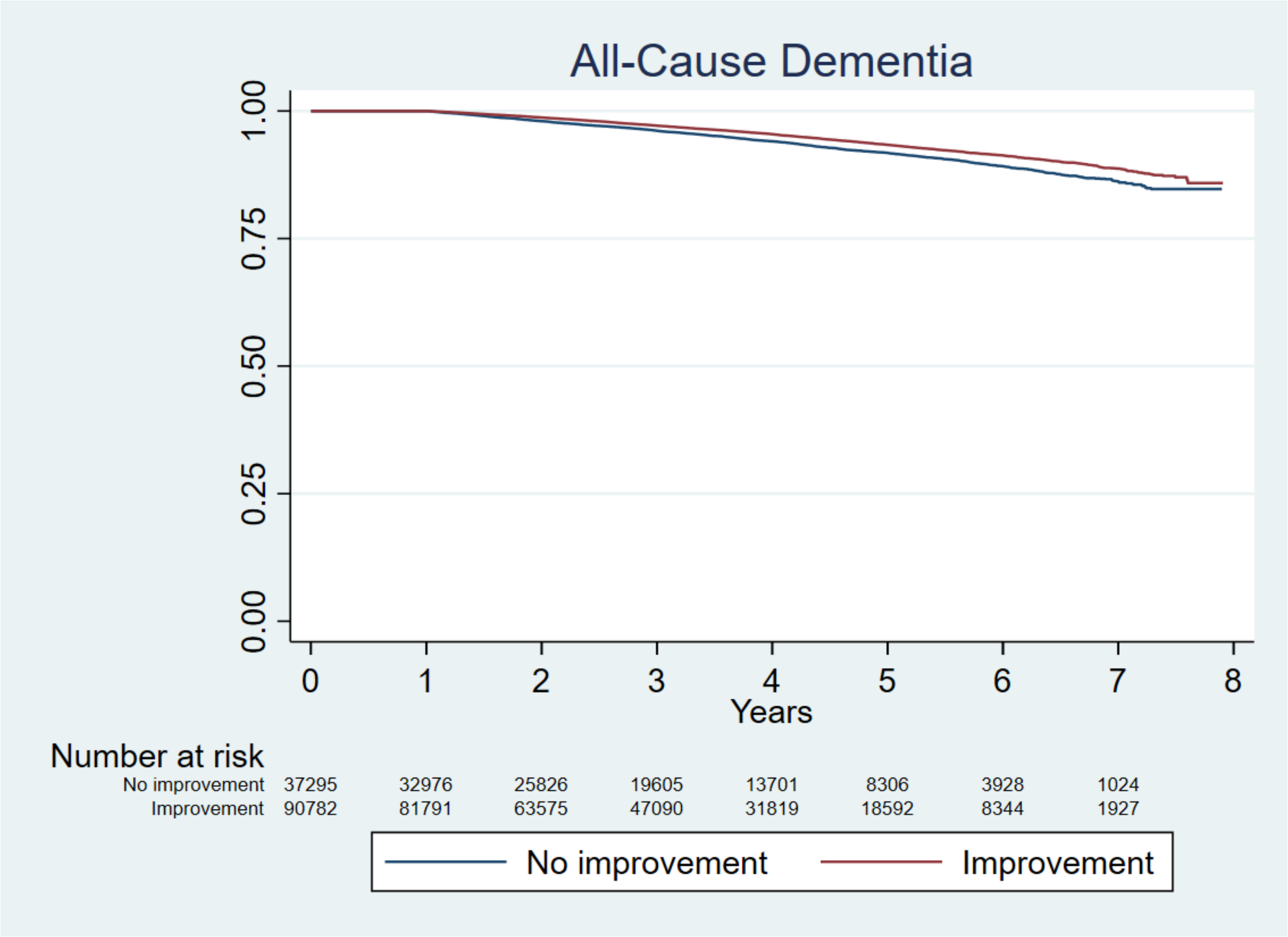

Supplementary Figure 3:

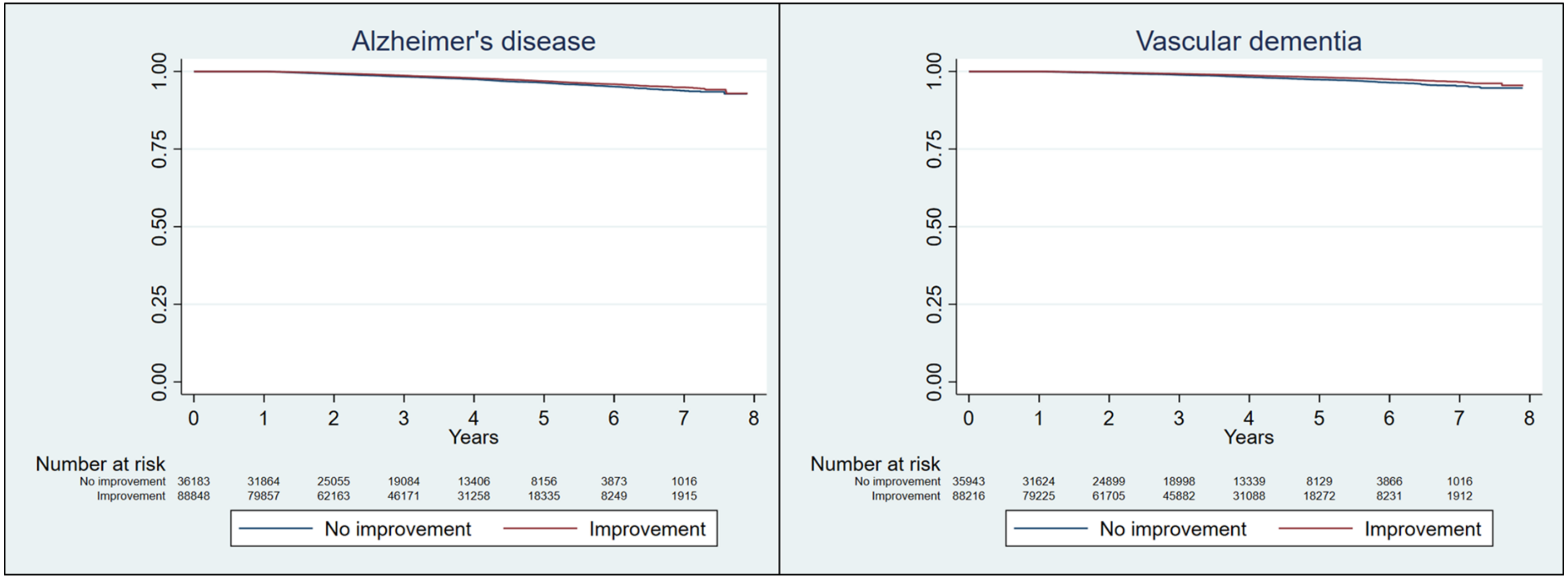

Supplementary Table 1: Thresholds for caseness and reliable change for Anxiety Disorder Specific Measures (ADSMs).

**Anxiety disorder**	**Specified Outcome Measure**	**Threshold for caseness**	**Threshold for reliable improvement**
Agoraphobia	Mobility Inventory	2.3	0.73
Health Anxiety	Health Anxiety Inventory	18	4
Obsessive compulsive disorder (OCD)	Obsessive Compulsive Inventory	40	32
Panic disorder	Panic Disorder Severity Scale	N/A	N/A
Post-traumatic stress disorder (PTSD)	Impact of Events Scale	33	9
Social anxiety disorder	Social Phobia Inventory	19	10

Supplementary Table 2: Characteristics of analytic sample by reliable improvement status.

	No reliable improvement (N=37,295)		Reliable improvement (N=90,782)	
	**N (%)**	**Mean (SD)**	**N (%)**	**Mean (SD)**
**Key variables**				
Dementia diagnosis				
No	35,377 (94·86)	-	87,273 (96·13)	-
Yes	1,918 (5·14)	-	3,509 (3·87)	-
Time to dementia diagnosis (Years)	-	3·10 (1·51)	-	3·16 (1·47)
**Demographic Covariates**				
Gender				
Male	11,516 (31·02)	-	28,069 (31·02)	-
Female	25,614 (68·98)	-	62,405 (68·98)	-
Age	-	71·65 (5·86)	-	71·52 (5·62)
Ethnicity				
White	31,696 (95·04)	-	79,529 (96·24)	-
Mixed	200 (0·60)	-	406 (0·49)	-
Asian	843 (2·53)	-	1,509 (1·83)	-
Black	337 (1·01)	-	699 (0·85)	-
Chinese	35 (0·10)	-	51 (0·06)	-
Other	239 (0·72)	-	445 (0·54)	-

IMD decile		-	5·83 (2·79)	-	6·05 (2·73)
**Clinical Covariates**					
Number of attended contacts		-	5·32 (4·06)	-	6·54 (3·98)
Taking psychotropic medications					
	No	14,851 (39·82)	-	37,263 (41·05)	-
	Yes	17,791 (47·70)	-	44,460 (48·97)	-
	Missing	4,653 (12·48)	-	9,059 (9·98)	-
Cardiovascular disease (at any time)					
	No	20,841 (55·93)	-	54,607 (60·19)	-
	Yes	16,420 (44·07)	-	36,113 (39·81)	-
Comorbid depression					
	No	7,055 (18·93)	-	20,763 (22·88)	-
	Yes	30,207 (81·07)	-	69,972 (77·12)	-
Baseline GAD-7 severity		-	13·38 (3·92)	-	14·19 (3·73)
Long-term health condition					
	No	12,185 (32·67)	-	34,213 (37·69)	-
	Yes	15,819 (42·42)	-	37,016 (40·77)	-
	Missing	9,291 (24·91)	-	19,553 (21·54)	-

Supplementary Table 3: Characteristics of analytic sample by dementia status.

	No dementia (N=122,650)		Dementia (N=5,427)	
	**N (%)**	**Mean (SD)**	**N (%)**	**Mean (SD)**
**Key variables**				
Anxiety reliable improvement				
No	35,377 (28·84)	-	1,918 (35·34)	-
Yes	87,273 (71·16)	-	3,509 (64·66)	-
Anxiety reliable recovery				
No	49,723 (40·58)	-	2,553 (47·14)	-
Yes	72,813 (59·42)	-	2,863 (52·86)	-
**Demographic Covariates**				
Gender				
Male	37,880 (31·00)	-	1,705 (31·54)	-
Female	84,318 (69·00)	-	3,701 (68·46)	-
Age	-	71·37 (5·59)	-	75·65 (6·41)
Ethnicity				
White	106,626 (95·87)	-	4,599 (96·42)	-
Mixed	588 (0·53)	-	18 (0·38)	-
Asian	2,280 (2·05)	-	72 (1·51)	-
Black	974 (0·88)	-	62 (1·30)	-

Chinese	84 (0·08)	-	2 (0·04)	-
Other	667 (0·60)	-	17 (0·36)	-
IMD decile	-	5·99 (2·75)	-	5·89 (2·80)
**Clinical Covariates**				
Number of attended contacts	-	6·21 (4·06)	-	5·59 (3·58)
Taking psychotropic medications				
No	50,189 (40·92)	-	1,925 (35·47)	-
Yes	59,555 (48·56)	-	2,696 (49·68)	-
Missing	12,906 (10·52)	-	806 (14·85)	-
Cardiovascular disease (at any time)				
No	73,674 (60·11)	-	1,774 (32·72)	-
Yes	48,886 (39·89)	-	3,647 (67·28)	-
Comorbid depression				
No	26,629 (21·72)	-	1,189 (21·94)	-
Yes	95,948 (78·28)	-	4,231 (78·06)	-
Baseline GAD-7 severity	-	13·97 (3·81)	-	13·57 (3·77)
Long-term health condition				
No	44,679 (36·43)	-	1,719 (31·67)	-
Yes	50,769 (41·39)	-	2,066 (38·07)	-
Missing	27,202 (22·18)	-	1,642 (30·26)	-

Supplementary Table 4: Cox proportional hazards models to test associations between reliable recovery from anxiety following psychological therapy and dementia incidence.

	**Model 1 (Unadjusted)**	**Model 2 (Adjusted for demographic factors)**	**Model 3 (Adjusted for demographic and clinical factors)**
Reliable recovery from anxiety	0.81 (0.77-0.85), <.0001	0.80 (0.75-0.84), <.0001	0.83 (0.78-0.88), <.0001
Gender	-	0.83 (0.78-0.89), <.0001	0.92 (0.86-0.98), .0067
Age	-	1.12 (1.12-1.13), <.0001	1.11 (1.10-1.11), <.0001
Ethnicity			
White	-	Ref	Ref
Mixed	-	0.67 (0.41-1.07), .094	0.68 (0.42-1.10), .113
Asian	-	0.90 (0.71-1.14), .389	0.93 (0.73-1.17), .537
Black	-	1.39 (1.07-1.79), .013	1.44 (1.11-1.86), .0056
Chinese	-	0.57 (0.14-2.29), .430	0.65 (0.16-2.59), .538
Other	-	0.64 (0.38-1.06), .080	0.67 (0.40-1.11), .123
Deprivation	-	0.98 (0.97-0.99), <.0001	0.99 (0.98-0.997), .015
Number of attended contacts	-	-	0.99 (0.98-0.99), .0011
Taking psychotropic medications			
No	-	-	Ref
Yes	-	-	1.19 (1.12-1.27), <.0001
Missing	-	-	1.18 (1.07-1.30), .0011
Cardiovascular disease			
No	-	-	Ref
Yes	-	-	2.00 (1.87-2.13), <.0001
Comorbid depression			
No	-	-	Ref

Yes	-	-	1.13 (1.05-1.22), .0018
Baseline anxiety severity	-	-	0.99 (0.98-0.99), .0015
Long-term health condition			
No	-	-	Ref
Yes	-	-	0.99 (0.92-1.06), .751
Missing	-	-	1.02 (0.94-1.11), .613

Supplementary Table 5: Cox proportional hazards models to test associations between reliable improvement in GAD-7 following psychological therapy and dementia incidence.

	Model 1 (Unadjusted)	Model 2 (Adjusted for demographic factors)	Model 3 (Adjusted for demographic and clinical factors)
GAD-7	0.81 (0.77-0.87), <.0001	0.81 (0.76-0.87), <.0001	0.85 (0.80-0.91), <.0001
Gender	-	0.83 (0.78-0.89), <.0001	0.92 (0.86-0.98), .014
Age	-	1.12 (1.12-1.13), <.0001	1.11 (1.11-1.12), <.0001
Ethnicity			
White	-	Ref	Ref
Mixed	-	0.70 (0.42-1.16), .167	0.72 (0.44-1.20), .210
Asian	-	0.87 (0.68-1.12), .284	0.89 (0.69-1.15), .386
Black	-	1.43 (1.09-1.88), .011	1.47 (1.12-1.94), .006
Chinese	-	0.85 (0.21-3.39), .814	0.94 (0.24-3.77), .934
Other	-	0.60 (0.34-1.05), .074	0.63 (0.36-1.11), .110
Deprivation	-	0.98 (0.97-0.99), <.0001	0.99 (0.98-0.998), .019
Number of attended contacts	-	-	0.99 (0.98-0.99), .002
Taking psychotropic medications			
No	-	-	Ref
Yes	-	-	1.18 (1.11-1.26), <.0001
Missing	-	-	1.17 (1.05-1.30), .004
Cardiovascular disease			
No	-	-	Ref
Yes	-	-	2.02 (1.88-2.16), <.0001
Comorbid depression			
No	-	-	Ref

Yes	-	-	1.12 (1.03-1.21), .005
Baseline anxiety severity	-	-	0.99 (0.98-0.998), .013
Long-term health condition			
No	-	-	Ref
Yes	-	-	0.99 (0.92-1.06), .732
Missing	-	-	1.01 (0.93-1.11), .752

Supplementary Table 6: Cox proportional hazards models to test associations between reliable improvement in anxiety following psychological therapy and dementia incidence, excluding dementia cases diagnosed within 2 years after IAPT.

		Model 1 (Unadjusted)	Model 2 (Adjusted for demographic factors)	Model 3 (Adjusted for demographic and clinical factors)
Reliable improvement in anxiety		0.83 (0.78-0.89), <.0001	0.86 (0.80-0.92), <.0001	0.89 (0.83-0.96), .0027
Gender		-	0.85 (0.79-0.92), <.0001	0.94 (0.87-1.01), .097
Age		-	1.12 (1.12-1.13), <.0001	1.11 (1.11-1.12), <.0001
Ethnicity				
	White	-	Ref	Ref
	Mixed	-	0.70 (0.41-1.21), .210	0.72 (0.42-1.24), .237
	Asian	-	0.93 (0.70-1.23), .603	0.96 (0.72-1.27), .754
	Black	-	1.50 (1.12-2.01), .007	1.56 (1.17-2.10), .003
	Chinese	-	0.82 (0.20-3.27), .777	0.94 (0.24-3.78), .935
	Other	-	0.61 (0.33-1.14), .123	0.66 (0.35-1.22), .183
Deprivation		-	0.97 (0.96-0.99), <.0001	0.98 (0.97-0.997), .014
Number of attended contacts		-	-	0.99 (0.98-1.00), .071
Taking psychotropic medications				
	No	-	-	Ref
	Yes	-	-	1.21 (1.12-1.30), <.0001
	Missing	-	-	1.17 (1.04-1.31), .008
Cardiovascular disease				
	No	-	-	Ref
	Yes	-	-	1.98 (1.84-2.14), <.0001
Comorbid depression				
	No	-	-	Ref

Yes	-	-	1.11 (1.01-1.22), .019
Baseline anxiety severity	-	-	0.99 (0.98-1.00), .183
Long-term health condition			
No	-	-	Ref
Yes	-	-	1.00 (0.92-1.08), .999
Missing	-	-	1.01 (0.92-1.11), .824

Supplementary Table 7: Cox proportional hazards models to test associations between reliable improvement in anxiety following psychological therapy and dementia incidence, excluding people taking psychotropic medications.

	**Model 1 (Unadjusted)**	**Model 2 (Adjusted for demographic factors)**	**Model 3 (Adjusted for demographic and clinical factors)**
Reliable improvement in anxiety	0.81 (0.74-0.89), <.0001	0.84 (0.76-0.93), .0011	0.87 (0.79-0.97), .012
Gender	-	0.79 (0.71-0.87), <.0001	0.87 (0.78-0.96), .007
Age	-	1.13 (1.12-1.14), <.0001	1.12 (1.11-1.12), <.0001
Ethnicity			
White	-	Ref	Ref
Mixed	-	0.52 (0.24-1.17), .114	0.51 (0.23-1.14), .100
Asian	-	0.92 (0.65-1.28), .611	0.91 (0.65-1.28), .592
Black	-	1.52 (1.07-2.16), .020	1.61 (1.13-2.29), .009
Chinese	-	1.06 (0.26-4.23), .938	1.10 (0.27-4.39), .897
Other	-	0.74 (0.37-1.48), .395	0.78 (0.39-1.56), .483
Deprivation	-	0.98 (0.96-0.996), .018	0.99 (0.97-1.00), .132
Number of attended contacts	-	-	0.99 (0.97-1.00), .067
Cardiovascular disease			
No	-	-	Ref
Yes	-	-	1.94 (1.74-2.16), <.0001
Comorbid depression			
No	-	-	Ref
Yes	-	-	1.25 (1.11-1.41), .0003
Baseline anxiety severity	-	-	0.99 (0.98-1.01), .238
Long-term health condition			
No	-	-	Ref

Yes	-	-	0.89 (0.80-0.99), .039
Missing	-	-	0.95 (0.82-1.09), .427

Supplementary Table 8: Cox proportional hazards models to test associations between completion of a course of treatment in IAPT (2+ sessions) and dementia incidence.

	**Model 1 (Unadjusted)**	**Model 2 (Adjusted for demographic factors)**	**Model 3 (Adjusted for demographic and clinical factors)**
Course of treatment	0.72 (0.68-0.76), <.0001	0.78 (0.73-0.82), <.0001	0.79 (0.74-0.83), <.0001
Gender	-	0.81 (0.76-0.86), <.0001	0.89 (0.84-0.94), <.0001
Age	-	1.12 (1.12-1.12), <.0001	1.11 (1.10-1.11), <.0001
Ethnicity			
White	-	Ref	Ref
Mixed	-	0.69 (0.44-1.08), .106	0.69 (0.44-1.08), .108
Asian	-	0.91 (0.74-1.12), .373	0.91 (0.74-1.11), .362
Black	-	1.36 (1.08-1.70), .008	1.42 (1.13-1.78), .002
Chinese	-	1.33 (0.55-3.20), .521	1.52 (0.63-3.66), .349
Other	-	0.75 (0.49-1.15), .180	0.78 (0.51-1.20), .256
Deprivation	-	0.98 (0.97-0.99), <.0001	0.99 (0.98-0.996), .005
Taking psychotropic medications			
No	-	-	Ref
Yes	-	-	1.14 (1.08-1.21), <.0001
Missing	-	-	1.17 (1.07-1.29), .0005
Cardiovascular disease			
No	-	-	Ref
Yes	-	-	2.11 (1.98-2.24), <.0001
Comorbid depression			
No	-	-	Ref
Yes	-	-	1.11 (1.04-1.19), .003
Baseline anxiety severity	-	-	0.99 (0.99-1.00), .076

Long-term health condition			
No	-	-	Ref
Yes	-	-	1.00 (0.94-1.07), .946
Missing	-	-	1.02 (0.95-1.10), .556

Supplementary Table 9: Cox proportional hazards models including all-cause mortality as an event and dementia diagnosis as censored observations.

	**Model 1 (Unadjusted)**	**Model 2 (Adjusted for demographic factors)**	**Model 3 (Adjusted for demographic and clinical factors)**
Reliable improvement in anxiety	0.79 (0.76-0.82), <.0001	0.80 (0.77-0.84), <.0001	0.84 (0.81-0.88), <.0001
Gender	-	0.62 (0.59-0.64), <.0001	0.71 (0.68-0.74), <.0001
Age	-	1.10 (1.10-1.11), <.0001	1.09 (1.09-1.09), <.0001
Ethnicity			
White	-	Ref	Ref
Mixed	-	0.75 (0.55-1.01), .061	0.74 (0.54-1.00), .053
Asian	-	0.63 (0.53-0.76), <.0001	0.63 (0.52-0.75), <.0001
Black	-	0.58 (0.45-0.76), <.0001	0.61 (0.47-0.79), .0002
Chinese	-	0.80 (0.36-1.78), .582	0.91 (0.41-2.02), .813
Other	-	0.56 (0.39-0.80), .002	0.57 (0.40-0.82), .002
Deprivation	-	0.95 (0.094-0.95), <.0001	0.96 (0.95-0.97), <.0001
Number of attended contacts	-	-	0.99 (0.98-0.995), .0004
Taking psychotropic medications			
No	-	-	Ref
Yes	-	-	1.13 (1.08-1.18), <.0001
Missing	-	-	1.14 (1.07-1.22), .0001
Cardiovascular disease			
No	-	-	Ref
Yes	-	-	2.65 (2.53-2.78), <.0001
Comorbid depression			

No	-	-	Ref
Yes	-	-	1.19 (1.12-1.25), <.0001
Baseline anxiety severity	-	-	1.00 (0.99-1.01), .903
Long-term health condition			
No	-	-	Ref
Yes	-	-	1.34 (1.28-1.40), <.0001
Missing	-	-	1.05 (0.99-1.11), .084

Supplementary Table 10: Mixed effects Weibull survival model testing associations between reliable improvement in anxiety and dementia incidence, including IAPT service delivering intervention as a random effect.

	**Model 1 (Unadjusted)**	**Model 2 (Adjusted for demographic factors)**	**Model 3 (Adjusted for demographic and clinical factors)**
Reliable improvement in anxiety	0.78 (0.74-0.83), <.0001	0.80 (0.75-0.85), <.0001	0.83 (0.78-0.89), <.0001
Gender	-	0.83 (0.78-0.88), <.0001	0.91 (0.86-0.97), .004
Age	-	1.12 (1.12-1.13), <.0001	1.11 (1.11-1.11), <.0001
Ethnicity			
White	-	Ref	Ref
Mixed	-	0.65 (0.40-1.05), .077	0.66 (0.41-1.07), .093
Asian	-	0.90 (0.71-1.14), .392	0.92 (0.72-1.17), .484
Black	-	1.39 (1.07-1.81), .013	1.44 (1.11-1.87), .006
Chinese	-	0.61 (0.15-2.45), .489	0.69 (0.17-2.76), .599
Other	-	0.68 (0.42-1.12), .128	0.72 (0.44-1.17), .186
Deprivation	-	0.98 (0.97-0.99), <.0001	0.99 (0.97-0.997), .012
Number of attended contacts	-	-	0.99 (0.98-0.996), .003
Taking psychotropic medications			
No	-	-	Ref
Yes	-	-	1.19 (1.11-1.27), <.0001
Missing	-	-	1.17 (1.06-1.30), .003
Cardiovascular disease			
No	-	-	Ref
Yes	-	-	1.99 (1.86-2.12), <.0001
Comorbid depression			
No	-	-	Ref

Yes	-	-	1.13 (1.05-1.22), .001
Baseline anxiety severity	-	-	0.99 (0.98-0.999), .035
Long-term health condition			
No	-	-	Ref
Yes	-	-	1.00 (0.94-1.07), .947
Missing	-	-	0.96 (0.88-1.04), .326

Supplementary Table 11: Cox proportional hazards models including categorical age bands.

		Adjusted for demographic and clinical factors
Reliable improvement in anxiety		0.82 (0.77-0.87), <.0001
Gender		0.93 (0.87-0.99), .016
Age		
	65-69	Ref
	70-74	2.08 (1.91-2.27), <.0001
	75-79	3.73 (3.41-4.07), <.0001
	80-84	5.93 (5.39-6.52), <.0001
	85+	8.27 (7.36-9.29), <.0001
Ethnicity		
	White	Ref
	Mixed	0.67 (0.42-1.08), .098
	Asian	0.92 (0.72-1.16), .471
	Black	1.39 (1.07-1.79), .012
	Chinese	0.66 (0.16-2.64), .555
	Other	0.72 (0.44-1.18), .196
Deprivation		0.99 (0.98-0.998), .023
Number of attended contacts		0.99 (0.98-0.99), .001
Taking psychotropic medications		
	No	Ref
	Yes	1.20 (1.13-1.28), <.0001
	Missing	1.19 (1.08-1.32), .0004
Cardiovascular disease		
	No	Ref

	Yes	1.99 (1.87-2.13), <.0001
Comorbid depression		
	No	Ref
	Yes	1.13 (1.04-1.22), .002
Baseline anxiety severity		0.99 (0.98-0.999), .037
Long-term health condition		
	No	Ref
	Yes	0.98 (0.92-1.05), .621
	Missing	1.02 (0.94-1.10), .633

Supplementary Table 12: Cox proportional hazards models including continuous PHQ-9 score.

		Adjusted for demographic and clinical factors
Reliable improvement in anxiety		0.84 (0.79-0.89), <.0001
Gender		0.92 (0.86-0.98), .008
Age		1.11 (1.10-1.11), <.0001
Ethnicity		
	White	Ref
	Mixed	0.68 (0.42-1.10), .115
	Asian	0.93 (0.73-1.17), .524
	Black	1.43 (1.11-1.85), .006
	Chinese	0.64 (0.16-2.56), .527
	Other	0.71 (0.44-1.16), .175
Deprivation		0.99 (0.98-0.998), .023
Number of attended contacts		0.99 (0.98-99), .001
Taking psychotropic medications		
	No	Ref
	Yes	1.18 (1.11-1.26), <.0001
	Missing	1.17 (1.06-1.29), .0013
Cardiovascular disease		
	No	Ref
	Yes	2.00 (1.87-2.13), <.0001
Comorbid depression		1.02 (1.01-1.02), <.0001
Baseline anxiety severity		0.98 (0.98-0.99), .001
Long-term health condition		
	No	Ref

Yes	0.99 (0.92-1.06), .727
Missing	1.02 (0.94-1.10), .648
